# Asymptomatic Malaria in Refugees Living in a Non-Endemic South African City

**DOI:** 10.1371/journal.pone.0107693

**Published:** 2014-09-19

**Authors:** Joyce M. Tsoka-Gwegweni, Uchenna Okafor

**Affiliations:** Discipline of Public Health Medicine, School of Nursing and Public Health, University of KwaZulu-Natal, Durban, South Africa; Université Pierre et Marie Curie, France

## Abstract

**Background:**

Asymptomatic malaria infection in refugees is both a threat to the lives of the individuals and the public in the host country. Although South Africa has been experiencing an unprecedented influx of refugees since 1994, data on malaria infection among refugees is lacking. Such information is critical since South Africa is among the countries that have planned to eliminate malaria. The objective of this study was to determine prevalence of asymptomatic malaria infection among a refugee population living in a city of KwaZulu-Natal province, South Africa.

**Methods and Findings:**

A survey was conducted on adult refugee participants who attended a faith-based facility offering social services in a city of KwaZulu-Natal province, South Africa. The participants were screened for the presence of malaria using rapid diagnostic tests and microscopy. Demographic data for the participants were obtained using a closed ended questionnaire. Data was obtained for 303 participants consisting of 51.5% females and 47.5% males, ranging from 19 to 64 years old. More than 95% of them originated from sub-Saharan African countries. Two hundred and ninety participants provided a blood sample for screening of malaria. Of these, 3.8% tested positive for rapid diagnostic test and 5.9% for microscopy. The majority of malaria infections were due to *Plasmodium falciparum*.

**Conclusions:**

The study confirms the presence of asymptomatic malaria infections among a refugee population residing in a city of KwaZulu-Natal province that is not endemic for malaria. The results have important implications for both public health and malaria control in South Africa, particularly since the country has decided to eliminate malaria by 2018. To achieve this goal, South Africa needs to expand research, surveillance and elimination activities to include non-endemic areas, particularly with high refugee populations. We further recommend use of powerful diagnostic tests such as PCR for these interventions.

## Introduction

Malaria infection in refugees poses a health risk for both the infected individual and the public in the host country [1]. Studies conducted in countries with a high influx of refugees like US, Canada and Australia have reported prevalence rates of 3–50% among refugees that come from malaria endemic countries particularly Sub-Saharan Africa (SSA) [2]–[6]. For this reason, these countries have mandatory programmes and guidelines for screening and treatment of malaria in refugees either pre-departure from their own countries or on arrival in the host countries [5], [7]–[10].

Since 1994 South Africa is among the top countries that experience a high influx of asylum seekers mainly from SSA. It is reported that the number of applications for refugee status that are processed by the Department of Home Affairs annually exceed 100 000. By law, refugees in South Africa are free to move around, work and access basic social services [11]. However, many are now confined to the poor areas in cities and towns due to challenges of unemployment, language, xenophobia, costs of private health care services and attitude of staff at public health facilities [12]. As a result they rely on faith-based or non-government organizations which offer limited social and health care services [13]–[14]. Screening for surveillance and treatment of malaria, and other communicable diseases is not included in health programmes offered by these aid organizations, particularly since they are mainly located in malaria-free areas.

South Africa is reported to have exceeded the target for MDG 6 and it is among 34 African countries that have planned to eliminate malaria by 2018 [15]–[16]. Although it is known that 20–80% of malaria cases in South Africa are imported, the available data on malaria comes only from malaria endemic areas [17]–[18] which are targeted for the elimination programme. Data on prevalence of malaria, and in particular on asymptomatic infections, among refugee populations living in South Africa is lacking. For South Africa to be successful in its efforts to eliminate malaria, research on malaria among refugees, is needed to support implementation of active surveillance (regular screening for malaria) and management of malaria. Such information is critical for various reasons. First, refugees originate from many African countries with stable malaria transmission and have developed some immunity to malaria. It is likely that some would harbour asymptomatic infections, as has been observed in other studies [2], [4]. If transmitted, these infections could be more virulent to local populations who would have acquired little immunity under the hypo-endemic seasonal malaria transmission that prevails in South Africa. Second, the areas where these refugees live are non-endemic to malaria and therefore are not targeted by malaria control and elimination activities of the malaria control programmes that are available in endemic areas [19]. Third, unconfirmed reports of malaria cases in non-endemic areas of KwaZulu-Natal have been cited, mainly in the major urban cities. This study sought to determine prevalence of asymptomatic malaria infection among a refugee population living in a city of KwaZulu-Natal province, South Africa. In this paper we share how this aim was achieved.

## Materials and Methods

A cross-sectional survey was carried out during September 2012 and September 2013. The participants recruited into the survey included all adult refugees over the age of 18 years who sought social and health services for various reasons at a faith-based centre located in the city of Durban, KwaZulu-Natal Province, South Africa. Ethical approval to conduct the survey was granted by the University of KwaZulu-Natal Biomedical Research Ethics Committee (REF 122/11). Each participant signed a written informed consent to answer a closed ended questionnaire that incuded demographic details and malaria history. The participants also provided a peripheral blood sample to screen for malaria using *SD Bioline* rapid diagnostic tests (Standard Diagnostics Bioline, Korea) and microscopy thick and thin smears. All participants that tested positive for malaria were treated on site with a recommended antimalarial as per national treatment guidelines. Data entry and analysis was carried out in Microsoft Excel and frequency tables were generated to show demographic information of the study participants and prevalence of malaria.

## Results

Data was obtained for 303 participants consisting of 51.5% females and 47.5% males aged 19 to 64 years old. Of these participants, 289 originated from 12 different SSA countries, excluding South Africa. More than half of them came from DRC followed by Burundi, Rwanda and Zimbabwe. When asked about previous infections with malaria 89.1%% of participants responded that they had previously been infected with malaria prior to entering South Africa (Table 1). Two hundred and ninety participants provided a peripheral blood sample for screening of malaria. The prevalence of asymptomatic malaria was 3.8% for RDT, 5.9% for thin blood smear and 4.5% for thick blood smear. The majority of malaria infections were due to *P. falciparum* (88.2%) and the remainder resulted from mixed infections of *P. falciparum*/*P. vivax* (5.9%) and *P. falciparum/P. ovale* (5.9%). A higher prevalence of malaria was observed in participants that were male, from DRC and Burundi, in the age group 21–30 year olds and had a secondary level of education (Table 1). It was not possible to indicate a statistical difference due to low numbers of infected participants.

**Table 1:** Malaria infection and participants characteristics.

Variable	Malaria status (%) *n = 17*	Total (%) *n = 303*
***Age***		
≤20	3 (17.6)	9 (3.0)
21–30	6 (35.3)	148 (48.8)
31–40	3 (17.6)	101 (33.3)
41–50	3 (17.6)	33 (10.9)
≥50	1 (5.9)	6 (2.0)
Unknown	1 (5.9)	6 (2.0)
***Gender***		
Female	5 (29.4)	156 (51.5)
Male	11 (64.7)	144 (47.5)
Unknown	1 (5.9)	3 (1.0)
***Education***		
None	1 (5.9)	15 (5.0)
Primary	2 (11.8)	67 (22.1)
Secondary	11 (64.7)	191 (63.0)
Tertiary	3 (17.6)	27 (8.9)
***Marital status***		
Married	8 (47.1)	177 (58.4)
Single	9 (52.9)	117 (38.6)
Other	0 (0)	8 (2.6)
***Occupation***		
Employed	8 (47.1)	180 (59.4)
Unemployed	9 (52.9)	114 (37.6)
Unknown	0 (0)	9 (3.0)
***Country of origin***		
DRC	8 (47.1)	154 (50.8)
Burundi	7 (41.2)	98 (32.3)
Rwanda	0 (0)	12 (4.0)
Zimbabwe	0 (0)	10 (3.3)
Other countries	2 (0)	14 (4.6)
Unknown	0 (0)	15 (5.0)
***Previous malaria infection***		
Yes	17 (100)	270 (89.1)
No	0 (0)	27 8.9)
Unknown	0 (0)	6 (2.0)

## Discussion

The results show that all, but two participants originated from SSA countries which are highly endemic for malaria transmission and carry a heavy burden of malaria [1], [20]. Similar to reports by the UNHCR [11] a large number of refugees come from the DRC, Burundi, Rwanda and Zimbabwe. Unlike in many studies conducted on refugees, particularly those from Asian countries, there is a slightly higher number of females than males in this population. The fact that many participants come from countries that experience stable malaria transmission explains why the participants did not present with symptoms of malaria when they were recruited into the survey. This is suggestive of some level of immunity acquired in the countries of origin. The possibility that they will thereby harbour undetected asymptomatic infections, makes them likely to act as a reservoir for transmission of malaria parasites to the South African population, who are generally non-immune because of the low seasonal transmission [16] putting them at risk of developing severe malaria.

The results confirm the presence of asymptomatic malaria (prevalence 3–5.2%) in a refugee population living in the city. This concurs with research from Canada, US, Australia and European countries that reported prevalence rates of 3–50% asymptomatic malaria among refugees entering these countries [2], [4], [7], [10]. It is possible that the prevalence in our sample could be under-estimation due to the low sensitivity of RDT and microscopy used to detect asymptomatic malaria infection. We believe that PCR tests would have yielded a much higher prevalence as was the case in other studies [21]–[24].

The presence of malaria transmission in an area classified as non-endemic for malaria raises two major concerns. First, this poses as a public health threat because malaria transmission may be re-introduced in the city. Malaria in South Africa is known to occur in the areas bordering Mozambique, Zimbabwe and Swaziland which lie in the north-eastern parts of KwaZulu-Natal, Limpopo and Mpumalanga provinces located mainly in rural areas [19]. Second, the presence of malaria transmission in the city is a major threat to tourism in the province and country as they are regarded as South Africa's premier tourist destinations. The Durban city is known as a popular tourism destination and a big event venue particularly for its warm sub-tropical climate even during winter. The vision of the tourism sector in KwaZulu-Natal province is to make the city globally renowned as Africa's top holiday destination by 2030 [25]. The city is currently marketed globally as a malaria-free tourist destination and the aim is to keep it under prevention of re-introduction (zero cases/1000 people) within the malaria elimination continuum [26]–[28]. In South Africa the most common vector is *Anopheles arabiensis*, but *Anopheles merus* has been on the increase. *Anophele funestus funestus* is another vector which re-appeared in the 2000's but was eliminated [17]. Maharaj et al agrees that the presence of these vectors in areas with malaria could lead to local transmission of malaria [17].

The results further demonstrate that two-thirds of infections are due to *P. falciparum*. This is expected given that almost all participants originated from SSA region which is highly endemic to *P. falciparum* malaria. Despite large numbers of SSA refugees that enter South Africa and the known fact that 20–80% of malaria in South Africa is imported, the national malaria elimination strategy is silent on how asymptomatic malaria will be dealt with [15], [26], [27]. Other countries known to have high influx of refugees from SSA have implemented mandatory screening and treatment programmes and guidelines of refugees when entering the country for the first time [7], [8], [10]. In South Africa it remains a challenge to introduce malaria screening at border areas and previous efforts proved unsuccessful [18], [27].

Though limited to a single setting, the results fulfill criteria used in other countries for enforcing treatment of asymptomatic malaria among refugees to prevent severe illness and transmission to local non-immune populations and re-introduction of malaria in areas declared malaria-free. We believe that our choice of the setting was appropriate because the city is one of the most populous areas and among the largest in the country with more than 3.5 million people and a high concentration of refugees [29]–[30]. Furthermore, given the challenges faced by refugees in South Africa especially the recent xenophobic attacks, refugees have limited access to public health facilities [12], [31]. They rely heavily on health care services offered by faith- and community-based organisations. We regard these facilities as reliable sources of data on refugees.

## Conclusions and Recommendations

To our knowledge the study is the first in South Africa to document the prevalence of asymptomatic malaria in a refugee population, residing in an urban area of KwaZulu-Natal province that is not endemic for malaria. These findings have important implications for both public health and malaria control in South Africa, particularly since the country has decided to eliminate malaria by 2018. To achieve this goal, South Africa needs to expand research, surveillance and elimination activities to include non-endemic areas and marginalized communities. The findings further emphasize the importance of integrating services such as malaria surveillance into other public health intervention programmes, and provide refugees with full access to public health services as prescribed by the law. It is envisaged that this study will serve as a basis for a comprehensive research on the burden of asymptomatic malaria among refugee populations residing in non-endemic areas of South Africa. Such research should include children and pregnant women, as well as using screening tests with high sensitivity for detection of low parasitaemia (PCR). The study presents further opportunities for research on the level of resistance to anti-malarials among refugee populations.
